# Xenon and modern anesthesiology: mechanism, clinical evidence and future development

**DOI:** 10.4103/mgr.MEDGASRES-D-25-00376

**Published:** 2026-05-15

**Authors:** Maria Magdalena Teper, Wiktor Perz, Bartosz Palacz, Lena Merchel, Natalia Marianna Kubiś, Karol Seweryn Błąd

**Affiliations:** Independent Public Health Care Institution of the Ministry of Interior Affairs and Administration in Kraków, Kraków, Poland; Collegium Medicum, Jan Kochanowski University of Kielce, Kielce, Poland

##  

Xenon has re-emerged as an important medical gas in contemporary anesthesiology due to its rapid onset, hemodynamic stability, and minimal systemic toxicity.^1^,^2^,^3^ Consistent results from preclinical and clinical studies show that xenon provides effective anesthesia while avoiding cardiovascular depression observed with conventional volatile agents.^4^,^5^ Its favorable pharmacokinetic profile, particularly its low blood-gas solubility, enables rapid titration and predictable emergence.^4^,^6^

It is well established that xenon acts through multiple molecular targets. A dominant and well-characterized mechanism involves inhibition of N-methyl-D-aspartate (NMDA) receptors, including competitive interaction at the glycine-binding site.^4^ Molecular dynamics simulations reveal at least three xenon-binding pockets in the NMDA receptor subunit 1 (GluN1) ligand-binding domain, supporting a structural basis for NMDA antagonism.^4^ Beyond NMDA modulation, xenon also suppresses hyperpolarization-activated cyclic nucleotide-gated 2 (HCN2) channels in thalamocortical neurons, reducing *I*_h_ current and altering excitability in pathways central to unconsciousness.^7^ These effects disappear in HCN2 mutant mice lacking cyclic nucleotide binding, indicating a direct mechanistic link to xenon-induced hypnosis.^7^

Xenon also has neuroprotective potential in models of hypoxic-ischemic injury, traumatic brain injury, and anesthetic-induced developmental neurotoxicity, involving reduced excitotoxicity, preserved dendritic structure, and improved mitochondrial stability.^5^,^8^,^9^ Although early clinical trials have confirmed feasibility and safety in neonates, additive neuroprotection remains to be fully demonstrated.^6^ These convergent findings suggest that xenon is a unique anesthetic with promising therapeutic applications, although the extent to which these preclinical effects translate into additive clinical neuroprotection remains to be established.

In this perspective, we argue that the unique biological effects of xenon cannot be adequately understood through isolated mechanistic pathways alone. Instead, we propose that xenon’s clinical potential emerges from the convergence of its molecular interactions, system-level effects, and context-dependent application. Our aim is not to comprehensively review the literature, but to critically interpret where current evidence is robust, where it remains incomplete, and how these distinctions should inform future research and clinical use.

**Mechanisms of xenon:** For conceptual clarity, it is essential to distinguish between mechanisms necessary for xenon-induced anesthesia and sedation, and those proposed to mediate neuroprotective or cytoprotective effects. While these processes may overlap at the molecular level, they are not necessarily equivalent or causally linked.

***NMDA receptor modulation:*** NMDA receptor inhibition is a principal mechanism by which xenon produces anesthesia and neuroprotection.^1^,^9^ Xenon suppresses NMDA-mediated currents through both competitive and non-competitive mechanisms, including displacement of glycine from its GluN1 co-agonist site and voltage-independent channel inhibition.^1^,^4^,^9^ Molecular dynamics studies have identified multiple xenon-binding pockets within the glycine-binding domain-clustered near residue F758-which explains the loss of xenon sensitivity in F758W/Y mutants despite preserved glycine affinity.^7^,^9^ By limiting NMDA-driven calcium influx, xenon reduces excitotoxicity in hypoxic-ischemic, traumatic, and developmental injury models.^4^,^8^,^9^ Preclinical studies consistently show decreased neuronal injury markers, preserved synaptic function, and reduced lactate dehydrogenase release under metabolic stress.^8^,^9^ Collectively, these data establish NMDA receptor modulation as a central element of xenon’s anesthetic and protective profile. Importantly, although NMDA receptor inhibition is central to xenon’s anesthetic action, its contribution to neuroprotection likely depends on context, timing, and injury-specific factors, and should not be assumed to be a direct extension of sedation.

***Effects on thalamocortical signaling and HCN2 channels:*** Thalamocortical pathways are essential for arousal and sensory relay, and xenon modulates these circuits by inhibiting HCN2 channels.^1^,^9^ By shifting *I*_h_ activation to more hyperpolarized potentials and reducing *I*_h_ amplitude, xenon decreases the excitability of thalamocortical neurons and limits cortical information flow.^7^,^8^ These effects require an intact cyclic nucleotide-binding domain, as HCN2 mutant mice lacking cyclic nucleotide binding show no electrophysiological response to xenon and fail to exhibit sedation.^7^ Thus, HCN2 modulation, alongside NMDA receptor inhibition, reduces thalamocortical drive and supports xenon’s anesthetic state. However, the relative contribution of NMDA receptor inhibition and HCN2 channel modulation to xenon’s clinical effects remains uncertain and may vary depending on experimental and clinical context (**Figure 1**).

**Figure 1:**
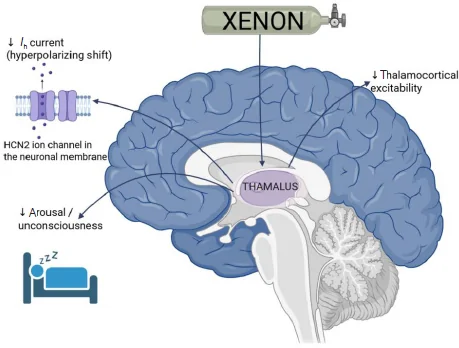
Mechanism of xenon on thalamocortical signaling. Created with BioRender.com. HCN2: Hyperpolarization-activated cyclic nucleotide-gated 2.

***Mitochondrial and cellular protective pathways:*** In contrast to the mechanisms primarily responsible for anesthesia and sedation, xenon’s mitochondrial and intracellular effects are predominantly discussed in the context of neuroprotection and cytoprotection, as xenon engages several intracellular pathways beyond its anesthetic targets that contribute to these protective effects.^3^,^9^ Mitochondria, which are highly vulnerable to anesthetic-induced dysfunction, represent a key site of xenon-mediated protection. Studies in neonatal and injury models show that xenon reduces activation of mitochondria-dependent apoptotic signaling, including suppression of caspase-3 activation and attenuation of oxidative stress-induced mitochondrial injury.^1^,^3^,^8^ Xenon also limits mitophagy dysregulation and preserves mitochondrial integrity in models of hypoxia, febrile seizures, and anesthetic-induced developmental neurotoxicity.^6^,^8^

In parallel, xenon enhances hypoxia-inducible factor-1α expression, even under normoxic conditions, promoting transcription of downstream genes involved in cellular recovery, angiogenesis, and metabolic adaptation. These effects align with improved neuronal survival in hypoxic-ischemic injury models.^6^,^8^ Xenon additionally preserves dendritic spine structure and stabilizes synaptic function in the presence of amyloid-β 1–42 and other toxic peptides, highlighting broader cytoprotective effects on neuronal architecture.^2^,^3^,^5^,^6^

Together, xenon’s mechanisms on mitochondrial stability, apoptotic signaling, hypoxia-inducible factor-1α activation, and dendritic maintenance form a complementary set of intracellular mechanisms that enhance its neuroprotective profile (**Figure 2**).^1^,^5^,^6^,^8^

**Figure 2:**
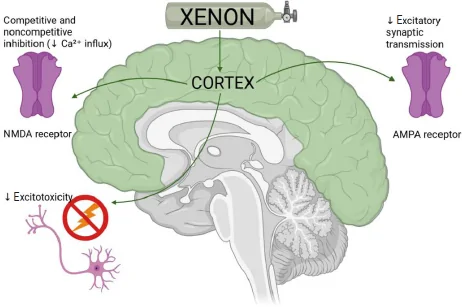
Xenon neuroprotective mechanisms in the cortex. Created with BioRender.com. AMPA: α-Amino-3-hydroxy-5-methyl-4-isoxazolepropionic acid; NMDA: N-methyl-D-aspartate.

***Additional emerging molecular targets:*** In addition to its established effects on NMDA and HCN2 channels, xenon interacts with several other molecular systems that likely contribute to its anesthetic and cytoprotective mechanisms.^6^,^8^ Xenon has been shown to inhibit α7 nicotinic acetylcholine receptors in a concentration-dependent, non-competitive manner, reducing excitatory cholinergic signaling implicated in arousal and stress responses.^6^,^8^ It also activates the two-pore domain Twike related potassium channel-1 (TREK-1), promoting membrane hyperpolarization and decreasing neuronal excitability-effects consistent with both anesthesia and neuroprotection.^3^,^6^,^9^ Preclinical data also suggest that xenon influences α-amino-3-hydroxy-5-methyl-4-isoxazolepropionic acid receptor-mediated transmission, reducing synaptic responses across multiple brain regions and contributing to attenuation of excitotoxicity. In addition, xenon enhances transcription of hypoxia-inducible factor-1α, broadening downstream cytoprotective signaling pathways.^3^,^6^,^8^ Recent work has raised the possibility that xenon may influence radical pair mechanisms or interact with cryptochrome-associated pathways. At present, however, these ideas remain speculative, lack direct experimental support in anesthetic or neuroprotective settings, and should be viewed as conceptual hypotheses rather than established contributors to xenon’s clinical effects.^6^,^8^ Together, these findings suggest that xenon acts through multiple pathways, some contributing to anesthesia and others more likely involved in cytoprotective signaling. However, the relative importance of these pathways and their translational relevance remain largely speculative.

**Clinical evidence for xenon in anesthesiology:**

***Fast, and potentially neuroprotective anesthesia:*** Randomized trials comparing xenon with volatile anesthetics-most often sevoflurane-consistently show that xenon provides equivalent anesthetic depth while delivering notably more stable intraoperative hemodynamics and faster early recovery.^5^,^6^,^10^ Several studies also report reduced postoperative elevations in neuronal injury biomarkers and signals toward improved early cognitive outcomes, though these findings remain inconsistent and underpowered.^5^,^6^,^8^ A recent meta-analysis suggests a possible neuroprotective advantage in selected populations, but the evidence is fragmented by small sample sizes and heterogeneous outcome measures.^5^ While these findings are intriguing, their translational relevance remains speculative in the absence of adequately powered and methodologically aligned clinical trials. The gap between robust preclinical neuroprotection and inconsistent clinical benefit likely reflects several translational challenges. Preclinical models typically involve well-defined injury timing, homogeneous subjects, and tightly controlled xenon exposure, whereas clinical populations are heterogeneous and perioperative exposures are shaped by variable comorbidity, concomitant anesthetics, and outcome definitions. In addition, cellular or histological neuroprotection observed in experimental models may not translate directly into short-term clinical endpoints, and biomarker changes may not reliably predict functional recovery.^4^,^6^,^8^ Together, these considerations support interpreting preclinical neuroprotection as proof of biological plausibility rather than a direct predictor of clinical efficacy.

From a broader perspective, current randomized controlled trials position xenon as at least non-inferior to sevoflurane, with plausible clinical advantages that have yet to be conclusively demonstrated.^6^,^8^ What is missing and urgently needed are larger, methodologically aligned trials capable of determining whether these early signals translate into meaningful patient benefit.

***Possible neurocognitive advantages:*** Evidence regarding xenon’s effects on postoperative neurocognition is mixed but suggests potential benefits in vulnerable populations.^2^,^6^,^8^ Several systematic review and meta-analysis reported lower postoperative levels of neuronal injury biomarkers after xenon anesthesia compared with sevoflurane, along with trends toward reduced early postoperative cognitive dysfunction.^5^,^11^ However, the overall certainty of evidence remains low due to small sample sizes, variability in cognitive testing protocols, and short follow-up durations.

Preclinical studies provide stronger mechanistic support for possible neurocognitive advantages. Xenon attenuates excitotoxic injury, preserves dendritic spine morphology, and stabilizes synaptic transmission in the presence of amyloid-β peptides changes closely linked to cognitive performance in models of neurodegeneration.^4^,^9^ Additionally, xenon’s modulation of HCN2 channels and NMDA receptors affects thalamocortical signaling patterns associated with attention and memory processing, offering further mechanistic plausibility.^4^,^7^

Although available clinical data do not yet demonstrate consistent cognitive superiority, in our view, the convergence of mechanistic plausibility and early clinical signals justifies continued investigation in high-risk surgical groups and pediatric populations.^1^,^5^,^9^

***Organ-protective properties in the perioperative period:*** Beyond its anesthetic effects, xenon demonstrates multi-organ protective properties in perioperative.^11^ Neurologically, xenon consistently attenuates excitotoxic injury, reduces oxidative stress, and limits activation of mitochondrial apoptotic pathways in models of hypoxic-ischemic injury, traumatic brain injury, and anesthetic-induced developmental neurotoxicity.^5^,^6^ These protective effects include preserved dendritic spine structure, improved mitochondrial stability, and reduced caspase-3 activation, collectively supporting enhanced neuronal survival during perioperative stress.^2^,^11^

Xenon’s modulation of HCN2 and NMDA receptors also contributes indirectly to neuroprotection by reducing pathological excitability and stabilizing thalamocortical and cortical networks, which may mitigate perioperative neuronal vulnerability, particularly in high-risk or aged populations.^7^,^11^

Organ protection extends beyond the central nervous system. Preclinical studies highlight xenon’s ability to reduce inflammation, preserve cellular energetics, and enhance hypoxia-inducible factor-1α signaling, mechanisms that may contribute to cardioprotection and improved tolerance to ischemia–reperfusion injury.^3^,^10^ Although robust clinical confirmation remains limited, these mechanistic and translational findings suggest, rather than establish, that xenon may represent a promising agent for reducing perioperative organ injury, particularly in procedures with ischemic risk.

***Safety profile and perioperative considerations:*** Xenon exhibits an excellent safety profile, characterized by hemodynamic stability, minimal respiratory depression, and lack of metabolic toxicity.^11^ Studies consistently report stable blood pressure and heart rate during xenon anesthesia, even in high-risk patients, reflecting its limited cardiovascular impact.^11^

Preclinical data show no long-term neurotoxicity at clinically relevant concentrations, although prolonged exposure in neonatal models may induce apoptosis, underscoring the importance of appropriate dosing and duration.^11^

Perioperatively, xenon’s rapid onset and offset facilitate predictable recovery, but its use is constrained by specialized delivery systems and high cost.^5^,^6^ Additionally, xenon’s limited availability and the need for closed-circuit equipment remain practical barriers to routine implementation.^5^,^6^ Despite these considerations, xenon remains a well-tolerated anesthetic with favorable safety characteristics when administered within established clinical parameters.

**Future directions:**

***Technological innovations:*** Recent technological advances have begun to reposition xenon from a technically demanding anesthetic to a more feasible clinical option.^3^,^5^ In particular, modern closed-circuit delivery systems, capable of efficiently recapturing exhaled xenon through real-time monitoring and automated flow control, have substantially reduced consumption and improved economic viability.^2^,^3^

Parallel progress in membrane-based separation, cryogenic extraction, and adsorption technologies suggests a future in which xenon can be recycled more effectively, potentially redefining cost barriers that have historically limited its use. The emergence of vaporizer-free delivery platforms and compact workstation integration further indicates a shift toward more practical perioperative deployment.^2^,^3^

Together, these innovations do more than refine existing techniques; they open the possibility that technology, rather than cost, will ultimately determine xenon’s place in modern anesthesia.

***Expanding clinical indications:*** Beyond its role as an anesthetic, xenon is increasingly emerging as a candidate therapeutic gas. Preclinical work consistently shows neuroprotection in hypoxic–ischemic encephalopathy, traumatic brain injury, and developmental neurotoxicity, suggesting that xenon may have value in perioperative neuroprotection and neurocritical care.^4^,^6^ Its combined mechanisms on NMDA and HCN2 signaling, along with stabilization of mitochondrial function, provide a coherent mechanistic framework for use in settings of acute neuronal stress.^4^

There is also growing interest in xenon’s cardioprotective potential during ischemia-reperfusion and major surgery. In neonates, xenon remains under evaluation as an adjunct to therapeutic hypothermia-an approach that is clearly safe, though not yet proven effective.^2^,^3^,^6^

Taken together, these developments point toward a future in which xenon could function not only as an anesthetic but as a broader cytoprotective agent. Whether this potential is realized will depend on larger, well-designed clinical trials.

***Research priorities:*** Despite strong mechanistic and preclinical evidence, xenon’s clinical potential remains constrained by gaps in translation. In our view, the most urgent need is for adequately powered, methodologically consistent randomized trials to determine whether xenon’s neuroprotective and organ-protective effects translate into tangible postoperative benefits, particularly in high-risk surgical, neonatal, and neurocritical care populations. Harmonizing dosing strategies, exposure durations, and cognitive outcome measures will be essential to overcome the heterogeneity that has limited prior studies.

Equally important is clarifying how NMDA, HCN2, TREK-1, and mitochondrial pathways interact to produce xenon’s multimodal profiles, and how these mechanisms vary across age and disease states. As technological advances make xenon delivery increasingly feasible, parallel cost–effectiveness analyses will determine whether broader clinical adoption is realistic.

Together, these priorities outline the key steps required to define xenon’s future role as both an anesthetic and a therapeutic agent.

**Conclusion:** Xenon occupies a unique position in modern anesthesiology as an anesthetic agent with a distinctive mechanistic profile and promising organ-protective properties. Its multimodal actions, including effects on NMDA receptors, HCN2 channels, TREK-1 signaling, and mitochondrial homeostasis, provide a strong biological rationale for benefits extending beyond anesthesia. While preclinical studies consistently support these mechanisms and clinical trials confirm feasibility and safety, clear superiority over standard volatile agents has not yet been demonstrated. From a clinical perspective, xenon should therefore be regarded as a context-sensitive anesthetic whose potential advantages are unlikely to be universal but may be clinically meaningful in selected high-risk populations. From a research perspective, priority should be given to conducting trials with sufficient statistical power and methodological consistency to establish associations between these well-defined mechanisms and patient-centered outcome measures.

*In preparing this work, the authors used ChatGPT for the purpose of improving language and readability. After using this tool, the authors have reviewed and edited the content as needed and accept full responsibility for the substantive content of the publication.*
